# Comparative Study of Anticipatory Postural Adjustments between Normal and Cervical Myelopathy Patients

**DOI:** 10.3390/jcm12103584

**Published:** 2023-05-21

**Authors:** Haruki Funao, Tatsuya Igawa, Masaru Matsuzawa, Norihiro Isogai, Ken Ishii

**Affiliations:** 1 Department of Orthopaedic Surgery, School of Medicine, International University of Health and Welfare, Chiba 286-8686, Japan; 2 Department of Orthopaedic Surgery, International University of Health and Welfare Mita Hospital, Tokyo 108-8329, Japan; 3 Department of Orthopaedic Surgery, International University of Health and Welfare Narita Hospital, Chiba 286-0124, Japan; 4 Department of Physical Therapy, School of Health Science, International University of Health and Welfare, Tochigi 324-8501, Japan; 5 Department of Orthopaedic Surgery, Keio University School of Medicine, Tokyo 160-8582, Japan; 6 Society for Minimally Invasive Spinal Treatment (MIST), Tokyo 101-0063, Japan

**Keywords:** anticipatory postural adjustment, cervical myelopathy, cervical spondylotic myelopathy, gait analysis, ossification of the posterior longitudinal ligament

## Abstract

Patients with cervical spondylotic myelopathy or ossification of the posterior longitudinal ligament have been considered to be prone to falls due to lower extremity dysfunction and gait instability. Anticipatory postural adjustments (APAs) are unconscious muscular activities to counterbalance perturbation. To date, there are no reports on APAs in cervical myelopathy patients, and quantification of postural control remains difficult. Thirty participants were enrolled, of which 15 were cervical myelopathy patients and 15 were normal age- and sex-matched controls. A three-dimensional motion capture system with force plates was used, and the APA phase was defined as the time between start of movement at the center of pressure and heel-off of the step leg. The APA phase (0.47 vs. 0.39 s, *p* < 0.05) and turning time (2.27 vs. 1.83 s, *p* < 0.01) were significantly longer, whereas step length tended to be shorter (305.18 vs. 361.04 mm, *p* = 0.06) in cervical myelopathy patients. There was a significant correlation between Japanese Orthopaedic Association lower extremity motor dysfunction scores and step length (*p* < 0.01). Cervical myelopathy patients are prone to falls due to longer APA phases with shorter step lengths. Analysis of the APA phase aids the visualization and quantification of postural control during initial gait in cervical myelopathy patients.

## 1. Introduction

Cervical myelopathy is a common disorder that results from spinal cord compression. The condition is caused by various conditions such as degenerative cervical spondylosis or ossification of the posterior longitudinal ligament (OPLL). Presenting symptoms for cervical myelopathy include motor weakness, sensory impairment, clumsy hands, gait disturbance, and bladder/bowel dysfunction. Patients with cervical myelopathy are at risk of falls not only due to weakness in the lower extremities but also by spastic gait or gait instability, and patients commonly experience fall-related deterioration of the symptoms [1].

According to data from the National Spinal Cord Injury Statistical Center, falls are the second most common etiology of spinal cord injury after vehicle accidents. The percentage of spinal cord injury due to falls increased from 16.5% of all spinal cord injuries in the 1970s (1972–1979) to 31.3% in recent years (2015–2021). Spinal cord injuries caused by falls are more frequent in the elderly. It was reported that the rates of fall-related spinal cord injuries were 7.9% for those under 15 years old, 10.5% between 16 and 30 years old, 22.9% between 31 and 45 years old, 38.7% between 46 and 60 years old, 53.4% between 61 and 75 years old, and 67.6% over 76 years old [2]. Spivak et al. [3] reported that cervical spine injury commonly occurs with minor trauma in patients over 65 years old and found that the mortality rate with associated spinal cord injury was 26%. This rate was 60 times higher than younger patients; moreover, return of functional motor recovery was delayed in older patients [3]. Because of the aging of the population, clinicians are expected to manage an increasing number of the elderly patients with cervical myelopathy who may potentially fall; therefore, the risk of falls in cervical myelopathy patients must be evaluated, especially in the elderly population.

At the onset of walking, the central nervous system employs two important components to control posture and restore equilibrium to prevent falls: anticipatory postural adjustments (APAs) and compensatory postural adjustments (CPAs) [4,5]. When postural perturbation is predictable, APAs are the first line of action to maintain a balanced posture for the perturbation, and CPAs, subsequently, start to act as a mechanism to complete the restoration of balance [6]. APAs are unconscious muscle activities to counterbalance the perturbation. Therefore, inadequate initial physical reaction due to impaired APAs may lead to falls. To date, impaired and altered APAs were reported in the elderly population and in those with Parkinson’s disease, stroke, and chronic low back pain [7,8,9,10,11,12,13,14]. To the best of our knowledge, there are no reports that investigated APAs in cervical myelopathy patients. Although there are many studies that investigated unconscious muscle activities using electromyography in the trunk and extremities for APAs analysis, the visualization and quantification of postural control remained difficult.

The aim of this study was to compare APAs between normal and cervical myelopathy patients using a three-dimensional (3D) motion capture system with force plates in order to visualize and quantify postural control during the initial step when making a turning motion.

## 2. Materials and Methods

**Figure 1:**
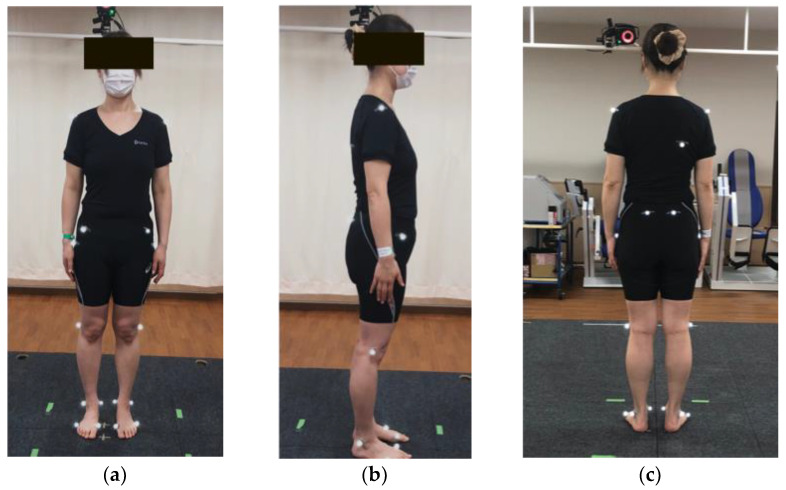
Reflective markers. Nineteen reflective markers. (**a**) Anterior view, (**b**) lateral view, and (**c**) posterior view. Markers were placed at the bilateral acromion processes, right angulus inferior scapulae, bilateral anterior superior iliac spine, bilateral posterior superior iliac spine, bilateral hips, bilateral lateral knees, bilateral lateral malleolus, bilateral medial malleolus, bilateral first metacarpophalangeal joints, and bilateral fifth metacarpophalangeal joints.

A total of 30 participants were examined, of which 15 patients (cervical myelopathy group, 8 males and 7 females) underwent cervical laminoplasty for cervical myelopathy due to cervical spondylotic myelopathy or cervical OPLL and 15 healthy age- and sex-matched volunteers (control group, 8 males and 7 females) were enrolled in this study. Cervical myelopathy was defined as the presence of compressive findings of the cervical spinal cord on magnetic resonance images and the Japanese Orthopaedic Association (JOA) score for cervical myelopathy of 13 points or less (Table 1). Patients with a history of cervical spine surgery or patients with severe cervical myelopathy who could not stand or walk without any assistance on a level were excluded due to the safety of the examination. Reflective markers were placed at 19 different points (Figure 1), and a 3D motion capture system consisted of 10 MX cameras (Vicon Motion Systems Ltd., Oxford, UK) and 6 force plates (AMTI, Watertown, MA, USA) that were used for gait analysis. The kinematic data were recorded at sample frequencies of 100 and 1000 Hz, and they were analyzed by Visual 3D analytical software (BodyBuilder3.6, Vicon Motion Systems Ltd., Oxford, UK).

**Figure 2:**
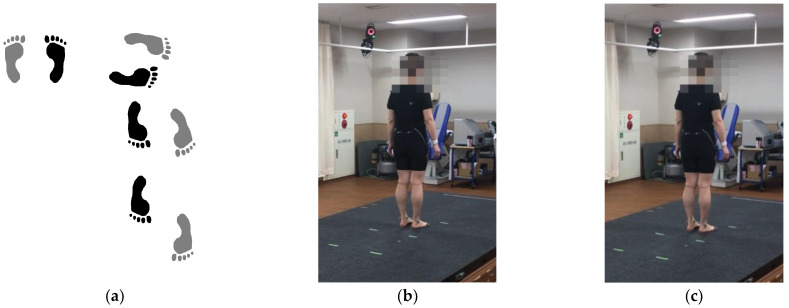

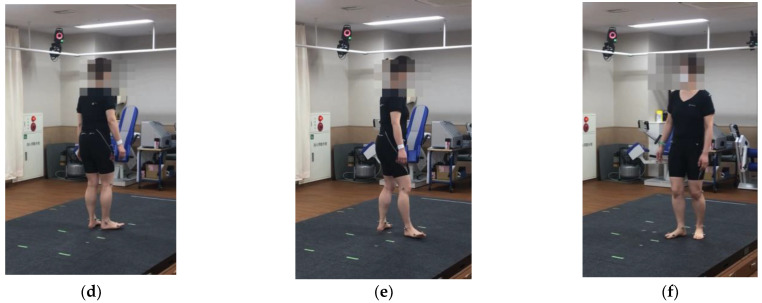
Gait analysis using a three-dimensional (3D) motion capture system with 6 force plates. (**a**) Both cervical myelopathy and control groups were required to turn around by side-step and walk. The APA phase, step time, turning time, step length, and % step length were measured dynamically using a 3D motion capture system with 6 force plates. (**b**) Initial movement at the center of pressure. The weight is evenly distributed on both feet. (**c**) The participant slightly shifts his/her weight on the right leg (step leg). (**d**) The timing of heel-off of the right leg (step leg). The participant places his/her entire weight on the left leg (pivot leg). (**e**) The timing of the participant taking the first step. (**f**) The timing of the participant turning his/her pelvis 180 degrees.

**Figure 3:**
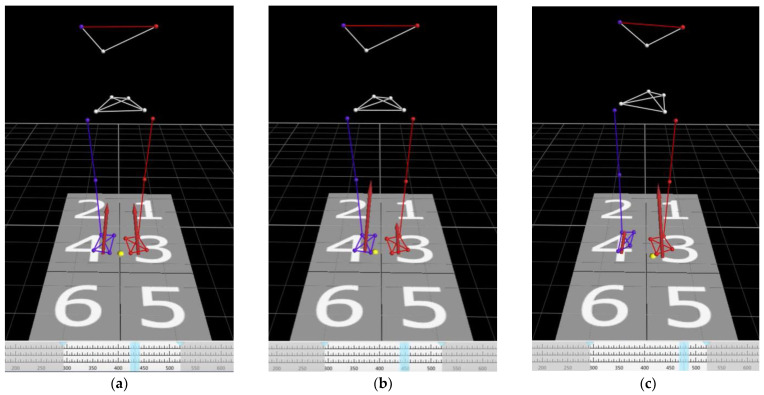

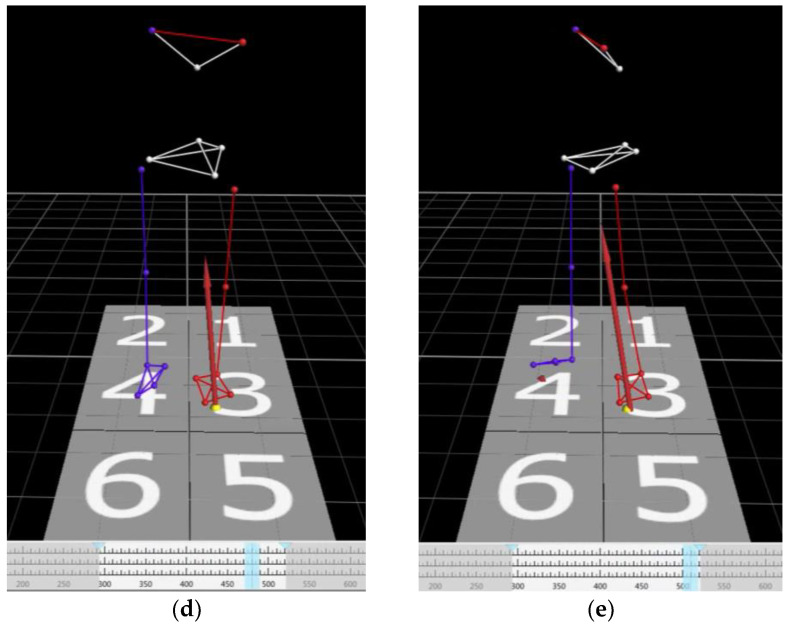
Time series of view of the markers and the center of pressure in 3D space. (**a**) Initial movement at the center of pressure. The weight is evenly distributed on both feet. (**b**) The weight is initially shifted on the right leg (step leg). (**c**) The weight is shifted on the left leg (pivot leg). (**d**) The timing of heel-off of the right leg (step leg). The participant places his/her entire weight on the left leg (pivot leg). (**e**) The timing of the participant taking the first step. The APA phase was defined as the time between starting of the movement of the center of pressure (COP) and heel-off of the right leg (step leg). Visualization and quantification of postural control can be achieved by analyzing the APA phase. Yellow dots: COP. Red arrows: ground reaction forces. Light blue lines: the timing of the images.

Both cervical myelopathy and control groups were required to turn around by side-step for the gait analysis (Figure 2a–f). The APA phase, step time, turning time, step length, and % step length were measured in both groups. The APA phase was defined as a time between the start of movement at the center of pressure (COP) and heel-off of the right leg (step leg) based on a previous report by Lyon and Day [15]. The APA phase could enable us to quantify postural control in side-step initiation (Figure 3a–e). Step time was defined as the time from when the step foot left the floor until it touched the ground again. Turning time was defined as the time from when the COP began to move until the pelvis turned 180 degrees. Step length was defined as the length of the first step of the step leg, and the % step length was calculated by dividing the step length by the patient’s height. % Step length = (step length/patient’s height) × 100%.

The JOA score for cervical myelopathy was evaluated in the cervical myelopathy group.

### Statistical Analysis

To evaluate the sample size, a preliminary study including 10 participants (cervical myelopathy group, *n* = 5; control group, *n* = 5) was performed using G* power software 3.1.9.4 (Heinrich Heine University, Düsseldorf, Germany) with an α-level of 0.05 and power of 0.85. The results of the power analysis showed that 30 participants (cervical myelopathy group, *n* = 15; control group, n = 15; effect size = 1.14) met the requisites, indicating a statistical significance.

All data were expressed as the mean ± standard deviation. Student’s *t*-test was used for comparing the APA phase, step time, turning time, step length, and % step length between the two groups. Correlations between the JOA scores and the APA phase, step time, turning time, step length, and % step length were determined using the Pearson correlation coefficient. SPSS, version 21.0 (IBM Corp., Armonk, NY, USA), was used to analyze the data. A *p*-value < 0.05 was considered statistically significant.

The study complied with the Declaration of Helsinki and was approved by the Ethics Review Committees (International University of Health and Welfare Mita Hospital, approval number: 5-9-21). Written informed consent was obtained from all participants in this study.

## 3. Results

The mean age was 70.0 ± 12.2 years old in the cervical myelopathy group and 70.2 ± 4.2 years old in the control group. The mean height/weight/body mass index were 160.7 ± 8.0 cm/59.6 ± 11.9 kg/22.9 ± 3.1 kg/m^2^ in the cervical myelopathy group, respectively, and 162.2 ± 10.1 cm/61.2 ± 13.3 kg/23.1 ± 3.6 kg/m^2^ in the control group, respectively. There were no significant differences in age (*p* = 0.95), height (*p* = 0.65), body weight (*p* = 0.74), and body mass index (*p* = 0.90) between the two groups.

The APA phase, step time, turning time, step length, and % step length were 0.47 ± 0.11 s, 0.32 ± 0.08 s, 2.27 ± 0.51 s, 305.18 ± 72.0 mm, and 18.94 ± 4.03% in the cervical myelopathy group, respectively, and 0.39 ± 0.08 s, 0.35 ± 0.08 s, 1.83 ± 0.29 s, 361.04 ± 81.9 mm, and 22.37 ± 5.52% in the control group, respectively (Table 2). The APA phase was significantly longer in the cervical myelopathy group (*p* < 0.05). Turning time was also significantly longer in the cervical myelopathy group (*p* < 0.01), whereas step length and % step length tended to be shorter in the cervical myelopathy group (both *p* = 0.06). There was no significant difference in step time between the two groups.

The mean JOA scores for upper extremity and lower extremity motor dysfunction were 2.27 ± 0.53 and 1.93 ± 0.56 points, respectively. The mean JOA scores for sensory dysfunction of the upper extremity, trunk, and lower extremity were 1.10 ± 0.43, 1.37 ± 0.48, and 1.17 ± 0.52 points, respectively. The mean JOA score for bladder dysfunction was 1.87 ± 0.52 points. In addition, the mean total JOA score was 9.67 ± 1.91 points in the cervical myelopathy group.

The correlation coefficients between the JOA scores and the APA phase, step time, turning time, step length, and % step length are shown in Table 3. There were significant correlations between JOA score of motor dysfunction of the lower extremity and step length and % step length. Although the total JOA score tended to have a correlation with step length, it did not reach a significance. There were no significant correlations between the other parameters and the JOA scores.

## 4. Discussion

Cervical myelopathy due to cervical spondylotic myelopathy and OPLL may both lead to gait disturbance, and patients are at risk of falls. Kimura et al. [1] reported that nearly 50% of cervical myelopathy patients who underwent surgery experienced at least one fall within one year before surgery, and 18% of the patients experienced a fall-related deterioration of motor deficits. In recent years, the aging of the population became a worldwide problem, and the issue was especially pronounced in Japan. The proportion of the elderly population in Japan was the highest in the world at approximately 28.4% of its total population in 2019, and it is expected to reach 33.3% in 2036 and 38.4% in 2065 [16]. Cervical myelopathy is one of the common degenerative spinal conditions in the elderly population, and the prevalence is expected to increase among the elderly [17,18]. As a result, clinicians are expected to manage an increasing number of elderly patients with cervical myelopathy who may fall. OPLL can also lead to cervical myelopathy due to spinal cord compression [19]. The incidence of OPLL was reported as 0.8–3.0% in Asians and 0.1–1.7% in Caucasians [20], and OPLL is most frequently diagnosed in the cervical spine [21,22]. Patients with cervical OPLL are also at risk of a cervical spinal cord injury, even after a minor fall. Onishi et al. [23] reported that risk factors for cervical spinal cord injury associated with OPLL were elderly and a mixed or segmental type of OPLL. Therefore, it is important for both patients with cervical spondylotic myelopathy and OPLL to prevent falls.

Humans control their posture by using their trunk and extremity muscles to restore equilibrium and prevent falls [4,5]. APAs are the first line of action to restore balance when the postural perturbation is predictable [6]. A previous study reported that APAs were delayed in older adults as compared to young adults [7,8]. It was also reported that APAs were impaired in neurological disorders. Rogers et al. [9] reported that Parkinson’s disease patients demonstrated a longer APA duration, longer time to first step onset, and slower step speed than controls. A longer APA duration in stroke patients was also reported [10,11]. Moreover, recent studies reported impaired or altered APAs in musculoskeletal diseases. Garcez et al. [12] reported that elderly patients who had chronic low back pain demonstrated impaired postural control and less efficient APAs. Takeuchi et al. [13] reported impaired APAs in patients with hip osteoarthritis. They found that hip osteoarthritis patients required a longer APA phase than control subjects; however, there was no significant difference in the total duration of the lateral step between the control and hip osteoarthritis group. Viton et al. [14] also reported altered APAs in patients who underwent total knee arthroplasty. They speculated that there were potential contributors to the differences in electromyography amplitudes in total knee arthroplasty patients, such as impaired neural activation or efforts to reduce stress on the involved knee joint. Interestingly, Gélat et al. reported that APAs were longer and larger for those who viewed pleasant images compared to unpleasant images, regardless of the duration of exposure to the images [24]. This suggests that APAs are controlled by a complex interplay of physical and emotional factors to restore balance.

**Figure 4:**
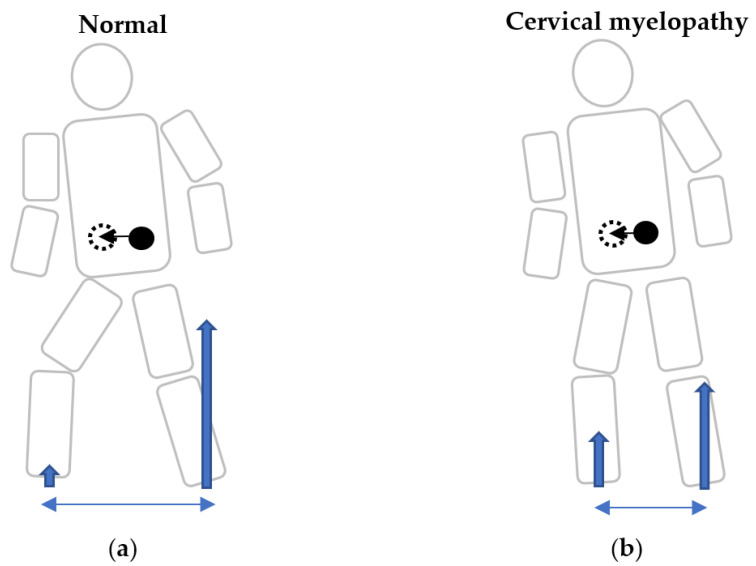
Schema of gait initiation at the same time period in normal subjects and cervical myelopathy patients. Schema of gait initiation in normal participants (**a**), and cervical myelopathy patients (**b**). A prolonged anticipatory postural adjustment phase represented a delay to shift the center of pressure of the pivot leg, resulting in a delay to free the step leg in cervical myelopathy patients. Because of an insufficient initial physical reaction, they may be prone to falls during gait initiation when making a turning motion. Blue arrows: ground reaction forces. Blue double arrows: step length. Black dots: center of mass (COM). Black dotted circle: predicted COM after the step.

To the best of our knowledge, there were no reports in the literature that investigated APAs in cervical myelopathy patients. Over the years, it was difficult to visualize and quantify the postural control and counterbalance the perturbation in cervical myelopathy patients. In the present age- and sex-matched study, we found that the APA phase (0.47 vs. 0.39 s) and turning time (2.27 vs. 1.83 s) were significantly longer, and step length (305.18 vs. 361.04 mm) and % step length (18.94 vs. 22.37%) tended to be shorter despite similar step time in cervical myelopathy patients compared to normal subjects. According to our results, patients with cervical myelopathy needed more time to counterbalance their postural perturbation with a longer APA phase. In addition, they exhibited a shorter step length and required more time to turn around. Therefore, patients with cervical myelopathy are prone to falls due to delayed counterbalance motion and shorter steps (Figure 4a,b). At gait initiation, the COP shifts on the pivot leg in order to free the step leg and generate the forward propulsive force [25]. A prolonged APA phase represented a delay to shift the COP of the pivot leg, resulting in a delay to free the step leg. This might lead to a shorter step and lesser forward propulsive force in the cervical myelopathy patient. The center of mass (COM) could be shifted to the intended direction with enough step length in a normal subject. In contrast, the COM might be shifted disproportionately with an insufficient step length in cervical myelopathy patients. Moreover, cervical myelopathy patients are not able to raise their legs adequately due to a delay to free the step leg. This can create a risk of stumbling on steps that may lead to falls.

In this study, patients with lower JOA scores in motor dysfunction of the lower extremity had a significantly shorter step length and % step length. Furthermore, the total JOA score tended to have a correlation with step length. However, other parameters including the APA phase were not significantly correlated with JOA scores. Possible reasons could be that the number of subjects was small, and it was difficult to perform the gait analysis in the patients with severe cervical myelopathy who could not stand or walk without any assistance due to a risk of fall during the examination. Another possibility was that the APA phase should be considered as a parameter separately from conventional outcome measurement for cervical myelopathy. The JOA scoring system for cervical myelopathy was developed by the Japanese Orthopaedic Association in 1976 [26]. The JOA score is widely used as a standard scoring system for cervical myelopathy all over the world. There were various outcome measurements for cervical myelopathy including the JOA scoring system, Nurick scoring system [27], neurosurgical cervical spine score [28], European myelopathy score [29], myelopathy disability index [30], and Cooper myelopathy scale [31]. However, these outcome measurements do not evaluate physical reaction during gait initiation in cervical myelopathy patients. Our results suggested that postural control in cervical myelopathy cannot be evaluated by conventional outcome measurement tools alone. Although Kimura et al. reported previous falls and a higher baseline of the 5-question Geriatric Locomotive Function Scale were predictors of postoperative recurrent falls [32], it could not quantify postural control in cervical myelopathy patients. In contrast, the APA phase can potentially be a useful quantitative parameter to evaluate counterbalance motion during gait initiation whether patients are at risk of falls. Therefore, it would be useful for clinicians to educate fall-prevention measures, recommend rehabilitation, and provide guidance on the use of orthosis or canes to those patients according to the impairment in the APA phase. Additionally, also, the APA phase would be pre- and postoperative outcome measurements in addition to the conventional evaluations for cervical spine surgery. Aruin et al. [6] recommended that APA-based interventions can be an effective rehabilitation approach in improving postural control, functional balance, mobility, and quality of life in individuals with balance deficit. APA-based interventions are rehabilitation approaches based on retraining the ability to generate and utilize APAs. Previous studies demonstrated that a four-week APA-based training program involving ball-catching and throwing activities resulted in improved clinical outcome measures of balance in older adults compared to control subjects who did not receive APA-focused training [33,34]. A recent study reported that virtual reality training may be a possible interventional strategy for enhancing the APAs of patients with chronic nonspecific low back pain [35]. These training or rehabilitation programs can potentially be a useful tool in patients with cervical myelopathy.

There were several limitations to the present study. First, this study was conducted with a relatively small number of the patients with cervical myelopathy, and these results must be evaluated in a larger sample size. Second, the gait analysis used in this study was difficult to evaluate in patients with severe cervical myelopathy who could not stand or walk without any assistance. The APA phase should be further evaluated in patients with severe myelopathy who require the use of walking aids. Finally, the gait analysis using 3D motion captures and force plates is cumbersome for daily clinical practice. Recent studies reported smartphone-based assessments for APAs [36,37]. However, it is currently impossible to quantify the APA phase without using 3D motion captures and force plates. The development of a simple and cost-effective measurement tool for the APA phase is needed for future research and clinical practice.

## 5. Conclusions

In cervical myelopathy patients, the analysis of the APA phase using 3D motion captures and force plates can visualize and quantify postural control during the initial gait when making a turning motion. Cervical myelopathy patients are prone to falls due to longer APA phases with shorter step lengths. They need more time to counterbalance the perturbation compared to normal subjects. The APA phase would be useful for evaluating the risk of falls and for developing fall-prevention measures in cervical myelopathy patients. Although the analysis of APAs in this study was based on a turning motion in side-stepping, future studies should evaluate APAs in a variety of situations that are susceptible to falls, such as when climbing a step or standing up from a chair.

## Figures and Tables

**Table 1:** The Japanese Orthopaedic Association (JOA) scoring system for cervical myelopathy.

Category			Score
Motor dysfunction			
Upper extremity			
	unable to eat with chopstick, spoon, or fork, and/or unable to fasten button of any size		0
	able to eat with spoon, or fork but not with chopsticks		1
	either eating with chopsticks or writing is possible but inadequate, and/or large button can be fastened		2
	either eating with chopsticks or writing is clumsy but practical, and/or cuff button can be fastened		3
	normal		4
Lower extremity			
	unable to stand and walk by any means		0
	unable to walk without a cane or other support on a level		1
	able to walk independently on a level, but needs support on stairs		2
	capable of fast but clumsy walking		3
	normal		4
Sensory dysfunction			
Upper extremity			
	apparent sensory disturbance		0
	minimal sensory disturbance		1
	normal		2
Lower extremity			
	apparent sensory disturbance		0
	minimal sensory disturbance		1
	normal		2
Trunk			
	apparent sensory disturbance		0
	minimal sensory disturbance		1
	normal		2
Bladder dysfunction			
	complete retention		0
	severe impairment (sense of retention, or dribbling)		1
	mild impairment (pollakiuria, or urinary hesitancy)		2
	normal		3

**Table 2:** The APA phase, step time, turning time, step length, and % step length.

	Cervical Myelopathy		Normal		*p*-Value
	Mean	SD	Mean	SD	
**APA phase (sec)**	0.47	0.11	0.39	0.08	<0.05
**Step time (sec)**	0.32	0.08	0.35	0.08	0.30
**Turning time (sec)**	2.27	0.51	1.83	0.29	<0.01
**Step length (mm)**	305.18	72.00	361.04	81.90	0.06
**% Step length (%)**	18.94	4.03	22.37	5.52	0.06

APA: anticipatory postural adjustment.

**Table 3:** Pearson correlation coefficients between the JOA scores and the APA phase, step time, turning time, step length, and % step length in cervical myelopathy patients.

	APA Phase	Step Time	Turning Time	Step Length	% Step Length
JOA U/E motor dysfunction	−0.03 (*p* = 0.92)	−0.18 (*p* = 0.52)	−0.32 (*p* = 0.24)	0.13 (*p* = 0.65)	0.12 (*p* = 0.67)
JOA L/E motor dysfunction	0.17 (*p* = 0.55)	0.21 (*p* = 0.45)	−0.35 (*p* = 0.20)	* 0.68 (*p* < 0.01)	* 0.68 (*p* < 0.01)
JOA U/E sensory dysfunction	0.02 (*p* = 0.94)	0.17 (*p* = 0.55)	−0.32 (*p* = 0.35)	0.38 (*p* = 0.16)	0.37 (*p* = 0.16)
JOA trunk sensory dysfunction	0.19 (*p* = 0.50)	−0.12 (*p* = 0.66)	0.18 (*p* = 0.51)	0.00 (*p* = 0.99)	−0.1 (*p* = 0.74)
JOA L/E sensory dysfunction	0.15 (*p* = 0.59)	0.15 (*p* = 0.60)	−0.10 (*p* = 0.73)	0.38 (*p* = 0.17)	0.33 (*p* = 0.24)
JOA bladder dysfunction	0.07 (*p* = 0.80)	0.33 (*p* = 0.23)	0.00 (*p* = 0.98)	0.11 (*p* = 0.70)	0.12 (*p* = 0.66)
JOA total	0.11 (*p* = 0.69)	0.12 (*p* = 0.66)	−0.27 (*p* = 0.34)	0.45 (*p* = 0.09)	0.40 (*p* = 0.14)

JOA: Japanese Orthopaedic Association, U/E: upper extremity, L/E: lower extremity, APA: anticipatory postural adjustment. * *p* < 0.01.

## Data Availability

Not applicable.
